# The Antitoxine Treatment of Diphtheria

**Published:** 1895-03

**Authors:** 


### EDITORIAL DEPARTMENT.

F. E. DANIEL, M. D., Editor.
S. E. HUDSON, M. D., Managing Editor.
A. J. SMITH. M. D., Galveston, Associate Editor.

EDITORIAL STAFF:

PROF. J. E. THOMPSON, M. D., Texas Medical College, Galveston; Surgery.
PROF. WM. KEILLER, M. D., Texas Medical College, Galveston; Obstetrics and Gynecology.
PROF. DAVID CERNA, M. D., Texas Medical College, Galveston; Therapeutics.
PROF. A. J. SMITH, M. D., Texas Medical College, Galveston; Medicine
DR. ISADORE DYER, Tulane University; Dermatology.
DR. R. H. L. BIBB, Saltillo, Mexico; Foreign Correspondent.

Official organ of the West Texas Medical Association, the Houston District Medical Association, the Austin District Medical Society, the Galveston County Medical Society, and several others.

### THE ANTITOXINE TREATMENT OF DIPHTHERIA.

The wonderful development of bacteriology within the last few years has certainly revolutionized medical science, and recent discoveries in that field seem to threaten the complete overthrow of every old system of therapeutics. In spite of the newer remedies, legion in number, which modern chemistry has elaborated and continues to elaborate, serum therapy to-day seems bent on changing materially the course of medical treatment at large. The chief claims of serum therapy appear to be directed to supersede the rational application of drugs from a knowledge of their physiological action. Whether serum therapy, an eminently scientific method of combatting disease, has come to stay, or whether it will eventually be substituted by a still later method, remains to be seen. The last word has certainly not been spoken by the tongue of bacteriological investigation.

Pasteur, Koch, and other modern investigators, whose brilliant labors have become classical in experimental medicine, have laid down principles of great scientific importance in the treatment of disease from the modern view of pathology, principles which, forming as they do an epoch in the progress of the healing art, are chiefly the outgrowth of bacteriological research. But it can not be gainsaid, indeed, that the modern treatment of disease has not come up fully to our expectations. In fact, the disappointment in the treatment of pulmonary tuberculosis by Koch's serum, following an unnecessary, untimely and unfortunate over-zealousness for the previous brilliant achievements of the German *savant*, is yet quite general. An undue exaggeration of Koch's claims, and not by that investigator himself (let it be said in all justice), but by others much less qualified in the matter, blinded for the time being the general good judgment of the medical profession, a circumstance which, sad to relate, soon led to disaster. Later investigations, however, made under calmer judgment, in Berlin, and particularly by Kitasato in Japan, appear to inspire to renewed hopes with regard to the therapeutic value of tuberculine in a modified form. Koch's battle, therefore, has not been entirely lost, apparently, and victory may yet be obtained with repeated combats by new forces under the direction of the same able German leader.

The treatment of diphtheria by a method identical to that of Koch in tuberculosis, is at present receiving a great deal of attention in the medical world, especially after the extensive experimental researches and clinical observations of Roux, embodied in a memoir read by this author at the recent Congress of Hygiene at Budapest. Serum therapy in the treatment of diphtheria is not an absolutely new discovery; nor does Roux himself, we believe, claim it as such. The treatment as recommended by Roux is new so far as its application to the human being is concerned. But many years ago Behring and Kitasato for the first time called the attention of the profession to the peculiar properties of the serum of animals immunized against tetanus and diphtheria. The researches of these two experimenters have been more or less confirmed by later observers, particularly in regard to the successful employment of tetanus antitoxine in the treatment of human lock-jaw.

The work of Roux, confirmatory to a large extent with respect to the use of serum in diphtheria, is so highly interesting from a scientific as well as from a practical point of view, that we propose to summarize, as briefly as possible, the essential portions of the subject under consideration. Following closely the work of Behring and Kitasato, Roux has tried, since 1891, in the treatment of diphtheria, an antitoxine serum, first upon the lower animals, and then, having ascertained its efficacy in these cases, on the human being. Accordingl to the results obtained, the mortality seems to have been reduced from 50 to 26 per cent, which is, to say the least, a very good showing.

The diphtheritic toxine is first prepared by cultivating the virulent bacillus of diphtheria in broth in the damp air. Flat bottomed flasks are used, in which is introduced a small quantity of alkaline broth, peptonized at 2 per cent; and are then placed in the incubator at a temperature of 37° C. A current of damp air is made to pass by means of a rubber tube, through a corresponding wash bottle. At about a month culture is sufficiently rich in toxines to be utilized. The cultures are then filtered through a Chamberland tube, and the clear liquid is placed in flasks well corked and kept in the dark. As a general rule, 1-10 of a c. c. is sufficient to kill, in the course of twenty-four hours, a guinea-pig weighing 500 grammes. To immunize animals the diphtheritic toxine is first mixed with one-third of its volume of Gram's solution*, and then injected as follows: A medium-sized rabbit can stand at first, without inconvenience, ½ c. c. of the mixture. The injection is repeated after a few days, and the process is kept up for several weeks, gradually increasing the amount of iodized toxine, or lessening the quantity of the iodine, until the pure toxine is introduced. The animals experimented upon must be weighed frequently in order to avoid a loss of weight; if the latter occur the injection must be suspended, otherwise a fatal termination will be the result.

Of all the animals used for this sort of experimentation, the horse appears to be the easiest to immunize, and to furnish, at the same time, the greatest amount of antidiphtheritic serum. In the horse, the toxine is injected under the skin of the neck, in gradually increasing doses. After about eighty-seven days, Roux has been able to inject into the jugular vein of a horse as much as 200 c. c. of diphtheritic toxine, without the animal experiencing any bad effects. The serum removed in this way from the horse, has a preventive power superior to 50,000; in other words, a guinea-pig weighing 500 grammes will resist an inoculation of ½ c. c. of a violent diphtheritic culture, if the animal have previously received a quantity of serum equal to 1-50,000 part of its weight. The milk of a well immunized cow is a good source of antitoxine, but, though it may render good service, such antitoxine is said to be less powerful than the serum. The serum obtained from the horse can be kept well, without undergoing any changes, in sterilized flasks to which a

---

*Gram's solution is composed as follows: Metallic iodine, 1 gramme; iodide of potassium, 2 grammes; distilled water, 300 grammes.—ED.

small piece of melted camphor has been added. The serum dried *in vacuo* can be sent to any distance, and resumes its preventive properties when dissolved in eight or ten volumes of sterilized water. The latter solution gives rise to a slight local swelling, an effect which does not seem to be produced by the natural serum.

The action of the serum in diphtheria of the mucous surfaces has been carefully studied by Roux through experimentation upon the lower animals, his observations being divided into four series, as follows: 1. *Serum injected as a preventive remedy.* Female guinea-pigs always resist the action of the virus when a sufficient amount of the serum is injected prior to the vaginal inoculation. The false membranes are formed it is true; but, compared with control experiments, the fever is less intense, and there is less redness, less swelling of the mucous membrane. By the second day, the local lesions diminish, and the mucous membranes begin to show a reparative action. The animals thus inoculated preventatively, recover, while those used in the control experiments die on the sixth day. 2. *Serum injected after inoculation.* After about twelve hours, when all the symptoms of diphtheria are well marked, an injection of the serum produces, shortly afterward, a rapid diminution of the symptoms, and a final cure. 3. *Action of the serum on animals whose tracheas are inoculated.* Guinea-pigs and rabbits inoculated with the diphtheritic bacilli through the trachea, die in from three to five days. But when thus injected after having received the serum, those animals exhibit no symptoms whatever of the disease. Again, the development of diphtheria is arrested if the serum be injected afterward. 4. *The serum in diphtheria complicated by the association of other bacteria.* The association of two kinds of microbes, diphtheritic bacilli and streptococci, produces in rabbits a diphtheria of rapid march, precisely as is observed in very young children. The symptoms and lesions are the same in both cases, and there appears a broncho-pneumonia, accompanied with an abundant bronchial secretion. In these cases, the serum rarely cures; not because there is an increase in the formation of diphtheritic toxines, or because the action of the antitoxine is hindered, but because the cells attacked by the streptococci no longer feel the stimulating action of the antitoxine. It appears, then, that in these cases larger amounts of the serum are required.

The results obtained by Roux in the treatment of diphtheria in the human being, are then described at length. He shows, in the first place, that during the years 1890, 1891, 1892 and 1893, of 3971 children admitted in the diphtheria ward of *L'Hospital des Enfantes Malades*, at Paris, 2029 died from the disease, giving a percentage of mortality of 51.71. From the first of February, 1894, to the time of preparing his communication, Roux applied the serum treatment on 448 children suffering from diphtheria in the same ward. Of this number 109 died, the percentage of mortality in this instance being only 24.25. All the conditions being the same, the difference in the two percentages speaks in favor of the new rreatment. The serum employed in the latter cases was obtained from immunized horses, its activity being between 50,000 and 100,000; that is, a guinea-pig receiving 1-50,000 part of its own weight of the serum, would support a few hours later, without inconvenience, a dose of the toxine sufficiently powerful to kill, in about thirty hours, another guinea-pig used in the control experiment.

In the children observed, the injection was made subcutaneously. It caused no pain in the majority of cases, and no local reaction. The injection was made preferably under the skin of the flank, the initial dose of the serum being 20 c. c. Twenty-four hours afterwards, another injection was made, the amount introduced varying from 10 to 20 c. c. The average weight of each one of the children treated was fourteen kilos (3½ pounds), so that in the first injection each little patient received about 1-1000 part of its own weight of the serum. The minimum dose of the antitoxine injected to each patient during the treatment, was 20 c. c., and the maximum 125 c. c. During convalescence, a few days after the administration of the serum, ill-defined eruptions appeared, resembling urticaria. These eruptions were not accompanied with any feverish reaction, and were evidently due to the antitoxine.

It is alleged that the general condition of the children treated as above described, improves rapidly, unless the disease is far advanced. The appetite soon returns, and the loss of weight is only slight. The false membranes cease to form in the twenty-four hours following the injection, and become completely detached in from thirty-six to forty-eight hours. The cervical ganglia remain engorged, but there is no infiltration of the cellular tissue surrounding them. The temperature is rapidly lowered under the action of the serum, the depression occurring generally on the day following the first injection. The fall of the temperature is sudden, and is usually a good sign. But if the temperature does not fall soon below 30° C., the prognosis is not so favorable, and it is then advised to repeat the injections of the serum. Compared with the action on the temperature, the pulse is but little influenced by antitoxine. The pulse never becomes normal before the temperature, but throughout the course of the treatment it is not so irregular. The serum prevents the action of the toxines upon the kidneys, and, probably on this account, considerably diminishes the amount of albuminuria.

Roux concludes his remarkable paper by pleading against a hasty surgical interference, even if the child exhibit a croupy breathing. The serum should be tried, and we ought to wait as long as possible. To enhance the action of the antitoxine, this local treatment is recommended: Two or three daily irrigations with simple boiled water, or, better still, with water containing 5 per cent. of Labarraque's solution; no swabbing with caustic or toxic substances, and no phenic acid, no corrosive sublimate, the accidental swallowing of which remedies implies danger.

Such is the work of Roux. Should his treatment prove a permanent success, his name shall be written with letters of diamond in the golden pages of history, as one of the great benefactors of mankind.

But (notwithstanding later researches with Koch's lymph), the great tuberculine failure haunts us still. The lesson we learned in that celebrated ridiculous craze, can not be forgotten so soon. Therefore, placed on our guard, advised by a calm judgment, but thoroughly unbiased, we patiently wait for further developments in the treatment of diphtheria with antitoxine before we can accept, with any degree of absoluteness, the claims of the great French investigator. For certainly, in this age of untrammeled scientific research, the *magister dixit* of old times is no longer tenable!

